# *Actinomyces* sp. detected by next-generation sequencing in paraffin-embedded, formalin-fixed tissues of a dog with severe panophthalmitis and periocular cellulitis

**DOI:** 10.1177/10406387251410586

**Published:** 2026-01-21

**Authors:** Charles-Antoine Assenmacher, Kathy Mou, Ganwu Li, Kimberly Hsu, Orhan Sahin, Stephen D. Cole

**Affiliations:** Department of Pathobiology, University of Pennsylvania School of Veterinary Medicine, Philadelphia, PA, USA; Veterinary Diagnostic Laboratory, College of Veterinary Medicine, Iowa State University, Ames, IA, USA; Veterinary Diagnostic Laboratory, College of Veterinary Medicine, Iowa State University, Ames, IA, USA; True North Veterinary Eye Care, Winnipeg, Manitoba, Canada; Veterinary Diagnostic Laboratory, College of Veterinary Medicine, Iowa State University, Ames, IA, USA; Department of Pathobiology, University of Pennsylvania School of Veterinary Medicine, Philadelphia, PA, USA

**Keywords:** *Actinomyces* sp, dogs, panophthalmitis, 16S rRNA metagenomics sequencing

## Abstract

A 9-mo-old, castrated male Saint Bernard dog was presented for evaluation of periorbital swelling, severe uveitis, and secondary glaucoma. Concurrently, chest radiographs had evidence of pneumonia. Enucleation was performed after failure of aggressive medical management. Histopathology of the globe confirmed severe necrosuppurative panophthalmitis and periocular cellulitis with myriad intra- and extracellular bacteria forming long filamentous chains. The bacteria were gram-positive and GMS-positive but acid-fast–negative. Next-generation sequencing (NGS) was performed on formalin-fixed, paraffin-embedded (FFPE) tissue from the eye. We identified a bacterium in the *Actinomycetaceae* family with a 100% BLAST match, suggestive of the previously described *Actinomyces catuli* strain (CCUG 41709). Clinical improvement followed enucleation and continued medical management, leading to reduction of the periocular swelling and resolution of the lung disease. Uveitis is common in dogs and is the most common cause of glaucoma. In many cases of bacterial uveitis, the exact bacterial organisms remain unknown if culture is not performed before fixation. *Actinomyces* sp. should be considered in patients with severe endophthalmitis or panophthalmitis, especially with evidence of systemic disease. NGS on FFPE samples may be a useful tool for identifying infectious organisms, especially in cases in which culture is not an option.

Uveitis is a common clinical presentation in veterinary medicine and one of the most common causes of blindness in dogs.^15^ Uveitis can result from a wide variety of causes, including traumatic, infectious, idiopathic, or immune-mediated processes, or may be a manifestation of systemic disease or neoplasia, with lymphoma diagnosed most frequently.^2,15^ The spectrum of pathogens implicated in uveitis is broad and includes, among others, viruses (e.g., canine distemper virus), bacteria (e.g., *Ehrlichia canis*, *Rickettsia rickettsii*), fungal or algal organisms (e.g., *Blastomyces* sp., *Prototheca* sp.), and parasites (e.g., *Dirofilaria immitis*).^5,15^

The genus *Actinomyces* comprises a diverse group of anaerobic or facultative anaerobic gram-positive, non–acid-fast, filamentous, rod-shaped bacteria.^12,20^ In animals (including humans), these organisms are predominantly found on mucosal surfaces, particularly in the oral cavity.^12,18^ Ocular manifestations of *Actinomyces* infection have been reported in both human and veterinary literature, ranging from keratitis (following corneal trauma or surgery) to more dramatic endophthalmitis and panophthalmitis.^7,17,19^ In humans, corneal or intraocular surgery are the most commonly suspected route of infection.^10,13^ Dental procedures have also been proposed as a source of infection, possibly through transient bacteremia following oral manipulation.^17^ In domestic species, traumatic implantation with foreign bodies or surgical instruments is often the suspected cause of infectious uveitis; dental procedures have also been proposed in a number of cases.^1,6,15,21^

Diagnosis of *Actinomyces* infections often relies on visualization of organisms in cytologic or histologic preparations and by anaerobic culture.^9^ Next-generation sequencing (NGS) technology enables affordable, high-throughput DNA sequencing in a non-targeted manner, in contrast to the targeted detection of specific pathogens offered by other molecular methods such as PCR testing.^22^ We describe a case of *Actinomyces* sp. causing severe panophthalmitis in a dog, diagnosed by combining histopathology and 16S rRNA metagenomic sequencing of formalin-fixed, paraffin-embedded (FFPE) tissue.

A 9-mo-old Saint Bernard dog was presented to an ophthalmologist with a 1-wk history of a cloudy and painful right eye. The initial clinical examination revealed severe uveitis with hypopyon and periocular cellulitis, as well as secondary glaucoma (IOP OD: 65 mm Hg; OS: 11 mm Hg). Visual function tests confirmed blindness in that eye. Blood work performed during the initial assessment—including a CBC, routine chemistry panel, SNAP 4DX (Idexx), and *Leptospira* witness test (Zoetis)—revealed no abnormalities. A urinalysis and thorough abdominal ultrasound were also within normal limits. A 1.5 × 5.5-cm focus of alveolar disease was noted in the ventral portion of the cranial segment of the left cranial lung lobe on chest radiographs, consistent with localized pneumonia, or (less likely) non-cardiogenic pulmonary edema or parenchymal hemorrhage. Coagulation tests were performed to rule out a clotting defect and were within normal limits (activated partial thromboplastin time = 104 s [RI: 72–102 s]; prothrombin time = 14 s [RI: 11–17 s]). Medical management of the ocular condition was attempted and consisted of a combination of topical (prednisolone acetate 1.0% q6h topically; ketorolac ophthalmic drops q6h topically; ofloxacin q6h topically; latanoprost 0.005% ophth q8h topically; dorzolamide HCl 2% ophth q12h topically; and Optixcare Plus [Aventix] q12h topically) and systemic medications (doxycycline 200 mg [3.6 mg/kg] q12h PO; gabapentin 500 mg [9 mg/kg] q8–12h PO; Rimadyl [carprofen; Zoetis] 112.5 mg [2 mg/kg, q12h PO]; and amoxicillin–clavulanate 1,250 mg [23 mg/kg] q12h PO). Given a lack of response to the medical management, persistent discomfort, and irreversible blindness of the eye, enucleation was recommended and performed 10 d after initial presentation to restore comfort. The eye was fixed in 10% neutral-buffered formalin for 2–3 d, processed routinely, and 5-µm sections stained with H&E for histologic evaluation.

Grossly, the globe was severely enlarged (~3-cm diameter) and required additional trimming to fit into regular processing cassettes. The cornea was severely thickened (3–4-mm thick) and opaque white to tan (**Fig. 1A**). On cut section, the globe was filled with abundant pale-yellow-to-tan gelatinous material (**Fig. 1B**). The periocular tissues were firm and discolored white to tan.

**Figure 1.**
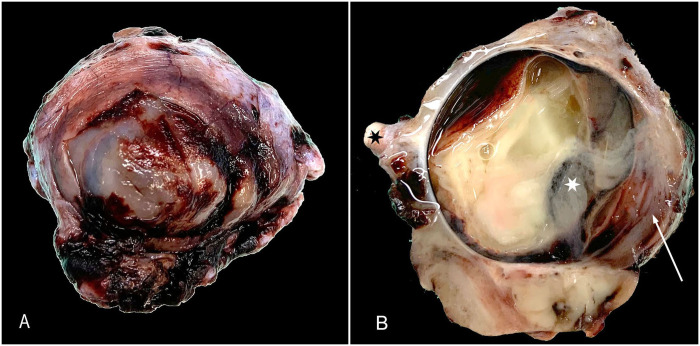
Gross appearance of the enucleated globe. **A.** The globe is severely enlarged, and the cornea is irregular and opaque white to tan. **B.** On cut section, the cornea (arrow) is severely thickened, and the globe is filled with pale-yellow-to-tan opaque material. The periocular tissues are discolored white to tan. The optic nerve (black star) and the lens (white star) are indicated.

Histologically, the extraocular tissues, the cornea, and all the intraocular tissues were diffusely expanded and partially effaced by a dense infiltrate of neutrophils, plus fibrin and necrotic debris (**Fig. 2A**). The cornea was also almost replaced by variably mature granulation tissue. Within the vitreous cavity, the inflammatory cell infiltrates surrounded aggregates of finely granular basophilic material, reminiscent of bacterial colonies (**Fig. 2B**). Scleral and periocular blood vessels were frequently necrotic or occluded by fibrin thrombi. The retina was diffusely detached, with subretinal accumulation of fibrin, protein-rich fluid, and hemorrhage. There was focal rupture of the posterior lens capsule with infiltration of neutrophils around the adjacent lens fibers. No signs of foreign material or trauma (i.e., bacterial implantation) were noted in the numerous sections examined. Histochemical stains for infectious organisms included MacCallum–Goodpasture Gram (**Fig. 2C**), Grocott–Gomori methenamine silver (GMS; **Fig. 2D**), and Ziehl–Neelsen acid-fast (**Fig. 2E**). These stains revealed numerous intra- and extracellular variably shaped gram-positive bacteria admixed with the inflammatory cell infiltrates in the vitreous cavity and periocular tissues. The bacteria, which often formed long filamentous extracellular chains, also stained positive with GMS but were acid-fast–negative. These morphologic and histochemical properties were suggestive of *Actinomyces* sp.

**Figure 2.**
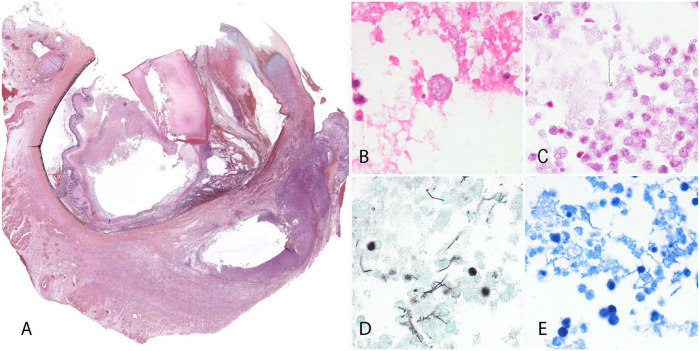
Histopathology of the globe. **A.** Subgross appearance of the globe. The extraocular tissues, the cornea, and all the intraocular tissues are diffusely expanded and partially effaced by a dense infiltrate of neutrophils, plus fibrin and necrotic debris. H&E. **B.** Higher magnification of the inflammatory cells within the vitreous cavity surrounding aggregates of finely granular basophilic material, reminiscent of bacterial colonies. H&E. **C–E.** The bacteria form long filamentous chains and are **C**) gram-positive and **D**) GMS-positive but **E**) acid fast–negative.

To confirm the identity of the infectious organisms and rule out other possible differentials, such as *Nocardia* sp.—and because no fresh tissue was available for culture—NGS was attempted on the FFPE tissues by performing bacterial 16S rRNA metagenomic sequencing analysis. Briefly, DNA was extracted (MagMAX FFPE DNA/RNA ultra kit; Applied Biosystems). For 16S library preparation, the V4 region of the 16S rRNA gene was sequenced (MiSeq sequencing platform, v2 MiSeq cartridges; Illumina) to produce 2 × 250-bp paired-end reads. DNA was amplified using the primer set 515f and 806r (forward V4: GTGCCAGCMGCCGCGGTAA; reverse V4: GGACTACHVGGGTWTCTAAT) with Schloss lab indices and AccuPrime Pfx SuperMix (Invitrogen). Library cleanup was performed (Agencourt AMPure XP beads; Beckman Coulter). Libraries were then quantified (1× dsDNA HS assay kit, 2.0 fluorometer; Qubit) and pooled to a single tube, with each library having equal final concentration. Taxonomies were assigned to amplicon sequence variants (**ASVs**) with a Naive–Bayes approach implemented in the classify-sklearn against the SILVA database. Phylogenetic trees for ASVs were generated using the align-to-tree-mafft-fasttree pipeline from the q2-phylogeny QIIME 2 plugin. Finally, stacked barcharts of taxa relative abundances were generated using qiime taxa barplot, and Krona plots were generated with Krona software.

The analysis of the 16S rRNA gene identified 170,259 reads, which resulted in 124,041 effective reads after data curation (**Fig. 3A**). Of those, 205 reads matched the genus *Actinomyces* (**Fig. 3B**). A 250-bp sequence from the reads had a 100% BLAST match with the previously described *Actinomyces catuli* strain 16S rRNA gene (CCUG 41709).^12^ The other bacterial organisms detected were predominantly gram-negative organisms; the few gram-positive bacteria detected (e.g., *Lactobacillus* sp., *Bacillus* sp.) did not share the morphologic and histochemical characteristics of the bacteria seen histologically in our sample. These bacteria likely were contaminants from the tissue sample or from the paraffin. Bacterial organisms from the skin or conjunctiva of the dog, for example, or from the trimming station or reagents are all known sources of contamination when working with FFPE samples.^3^

**Figure 3.**
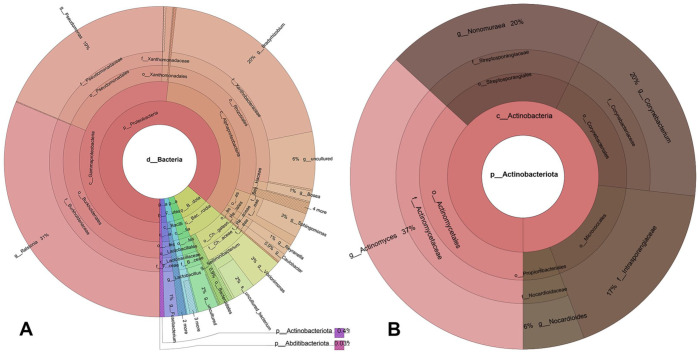
Krona plot of 16S rRNA metagenomic sequencing results from formalin-fixed, paraffin-embedded eye tissue. **A.** Numerous bacterial taxa were found within the sample. **B.** Among these, 205 reads were assigned to the genus *Actinomyces* (coral sectors).

Periocular swelling dramatically improved in the days following enucleation and continued medical therapy. The amoxicillin–clavulanate was increased to 1,500 mg (27 mg/kg) q8 PO following enucleation because of the suspicion of *Actinomyces* sp. infection and was discontinued after 3 mo. Follow-up bloodwork and chest radiographs were performed 4 wk after enucleation; lung disease had resolved. No recurrence of disease was detected at a final recheck 5 mo after enucleation.

The use of NGS on FFPE allowed us to identify *Actinomyces* sp. as the most likely organism at the genus level. *Actinomyces* sp. are ubiquitous gram-positive bacteria present in the soil and are part of the normal microbiota of humans and animals.^12,18^ These bacteria are found mostly on mucosal surfaces and are occasionally associated with disease, causing local infections and abscesses in the oral cavity, or distant infections following aspiration, traumatic penetration or, less likely, hematogenous spread.^12,18^ *Actinomyces* sp. bacteria have been isolated from cases of keratitis and endophthalmitis, often following corneal or intraocular surgery, or oral procedures.^10,13,17–19,21^ Hematogenous spread is suspected in dental-procedure cases because of the displacement of oral bacteria.^17,21^

Interestingly, a similar case of *Actinomyces* sp. endophthalmitis was reported in a dog with concurrent signs of pneumonia.^1^ In that dog, a vitreous paracentesis and transtracheal wash confirmed the endophthalmitis and pneumonia and revealed intra- and extracellular mixed bacteria in the vitreous, including long filamentous bacteria. A culture of both samples confirmed the presence of *Actinomyces* sp.; however, further speciation was not performed. Traumatic inoculation by a penetrating corneal foreign body (and subsequent hematogenous spread to the lung) was speculated in that case; however, an opposite scenario, with the pneumonic agent spreading to the anterior uvea, could not be excluded. Similarly, we could not confirm the pathogenesis of infection in our case. No prior oral procedures were reported in either case; however, aspiration of oral contents may have led to the pneumonia, followed by hematogenous spread to the uvea.

Unfortunately, the pneumonia in our case was not investigated further. Therefore, we cannot definitively connect the uveitis with the lung changes that were detected radiographically, apart from their similar response to antimicrobial therapy. Although we highly suspected *A. catuli* in our case based on the 100% BLAST match of the 16S rRNA fragment sequenced, the short length of that fragment (250 bp) made it difficult to rule out other *Actinomyces* sp. Reliable species-level identification typically requires sequencing of longer segments of the 16S rRNA gene; additional, more variable reference genes (such as *rpoB* or *gyrB*); or obtaining a draft genome sequence for average nucleotide identity analysis.^14^ This was not possible, however, given the short fragment available in our case, and the multiple bacterial organisms found in our sample. The treatment of choice for *Actinomyces* sp. is typically high-dose penicillin therapy, and identification to the species level is usually not clinically necessary.^20^ Importantly, with sequencing, we were able to rule out *Nocardia* sp., which would have required a different therapeutic approach.

The use of NGS has historically been restricted to fresh-frozen samples given the deleterious effects of fixatives on DNA and RNA, as well as the potential contamination during FFPE processing.^8,11^ In recent years, numerous studies have demonstrated the feasibility and usefulness of NGS on FFPE samples.^8,11,16^ Cost and limited access to NGS are significant hurdles to its routine use in clinics.^8^ Nonetheless, NGS can be a useful tool to identify bacterial organisms after tissue fixation, when culture is no longer available, and bacterial morphology (as in our case) can suggest clinical relevance of identified bacteria. A 2024 review highlighted the many benefits of NGS in veterinary medicine, not only in the context of infectious diseases, but also for metabolic diseases or for oncologic purposes.^4^ Although histology offers useful clues for identifying *Actinomyces* sp. in tissues, culture typically confirms the identity of those infectious organisms, as in cases of eye infections in dogs and cats.^1,19,21^ NGS becomes relevant when culture is no longer an option.
